# Reversible pH‐Responsive Coacervate Formation in Lipid Vesicles Activates Dormant Enzymatic Reactions

**DOI:** 10.1002/anie.201914893

**Published:** 2020-02-26

**Authors:** Celina Love, Jan Steinkühler, David T. Gonzales, Naresh Yandrapalli, Tom Robinson, Rumiana Dimova, T.‐Y. Dora Tang

**Affiliations:** ^1^ Max Planck Institute of Molecular Cell Biology and Genetics Pfotenhauerstraße 108 01307 Dresden Germany; ^2^ Cluster of Excellence Physics of Life TU Dresden 01602 Dresden Germany; ^3^ Center for Systems Biology Dresden Pfotenhauerstraße 108 01307 Dresden Germany; ^4^ Max Planck Institute of Colloids and Interfaces 14424 Potsdam Germany

**Keywords:** coacervates, liquid–liquid phase separation, microfluidics, pH responsive, protocells

## Abstract

In situ, reversible coacervate formation within lipid vesicles represents a key step in the development of responsive synthetic cellular models. Herein, we exploit the pH responsiveness of a polycation above and below its pK_a_, to drive liquid–liquid phase separation, to form single coacervate droplets within lipid vesicles. The process is completely reversible as coacervate droplets can be disassembled by increasing the pH above the pK_a_. We further show that pH‐triggered coacervation in the presence of low concentrations of enzymes activates dormant enzyme reactions by increasing the local concentration within the coacervate droplets and changing the local environment around the enzyme. In conclusion, this work establishes a tunable, pH responsive, enzymatically active multi‐compartment synthetic cell. The system is readily transferred into microfluidics, making it a robust model for addressing general questions in biology, such as the role of phase separation and its effect on enzymatic reactions using a bottom‐up synthetic biology approach.

## Graphical abstract

**The pH responsiveness** of a polycation above and below its pK_a_ is used to drive liquid–liquid phase separation to form coacervate droplets within lipid vesicles. This is reversible as the coacervate droplets can be disassembled by increasing the pH above the pK_a_. pH‐triggered coacervation in the presence of low concentrations of enzymes activates dormant reactions as the local enzyme and substrate concentrations are increased in the coacervate droplets which concomitantly changes the local environment of enzymes and reactants.

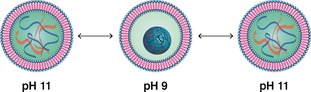

## Introduction

Hybrid protocells with structural features of both membrane‐bound and membrane‐free compartmentalization, which respond to external stimuli, offer a blueprint for the production of dynamic synthetic cellular models. These provide distinct, unique, and dynamic environments for the spatial organization of reactions. Membrane‐free coacervate microdroplets form by liquid–liquid phase separation between oppositely charged polypeptides, polynucleotides, polymers, and macromolecules. This coacervation process has attracted a lot of attention as it has recently been demonstrated to be one of the driving forces of cellular condensate formation.^1^, ^2^, ^3^, ^4^ Cytoplasmic phase de‐mixing^1^, ^2^ and synthetic liquid droplets^3^, ^4^, ^5^ have been shown to respond reversibly to changes in pH, salt, enzymes, and light in vivo and in vitro. Here, regulation of the molecular charge or chemical structure of the coacervate‐forming components leads to the mixing and de‐mixing of the microdroplet resulting in dramatic changes in the local environment.

Furthermore, hybrid protocells of membrane‐free sub‐compartments within either water‐in‐oil (w/o) emulsions, giant vesicles^6^ or proteinosomes have been formed by spontaneous self‐assembly methods in bulk or by using microfluidic methodologies.^7^, ^8^, ^9^ In these systems, changes in temperature or osmotic pressure from the exterior of the lipid vesicles have led to phase separation of aqueous two phase systems^10^, ^11^ and coacervates^12^ within the lipid vesicle. However, to the best of our knowledge there have been no examples of in situ formation of enzymatically active coacervates within lipid vesicles by an external pH stimulus.

To this end, we have developed a methodology for the in situ, pH‐reversible formation of coacervate microdroplets within giant unilamellar vesicles (GUVs). The lipid vesicle supports the isolation of the coacervate components from its exterior while permitting the transfer of water and protons across the lipid membrane.^13^ By exploiting the pH responsiveness of polylysine (PLys), a coacervate‐forming polypeptide,^3^, ^14^ we demonstrate that coacervate formation can be initiated within the lipid vesicle by an external change in pH. Above the p*K*_a_ of PLys, coacervate formation with a counter polyanion is arrested and below the p*K*_a_ of PLys, coacervate formation is triggered. We further show that this methodology is compatible with the encapsulation of enzymatic reactions and that in situ coacervate formation leads to the activation of enzymatic reactions by the increase in concentration and changes the local environment of the enzyme and reactants within a coacervate droplet. Our modular approach is robust, reproducible, and can also be transferred to microfluidic methodologies, further demonstrating the versatility of the method.

It is not completely understood why membrane‐free compartmentalization is important in biological systems. It has been postulated that these dynamical liquid–liquid phase separation processes can play a role in regulating biochemical processes.^15^, ^16^, ^17^, ^18^ However, investigating these processes within the complex environment of the cell is challenging. Our work therefore offers key steps in the synthesis of dynamic protocellular systems in bottom‐up synthetic biology and can be used to help test current models of the role of liquid–liquid phase separation in biology. Overall, this study presents a minimal model system for probing general phenomena in modern biology, where it is known that pH changes can lead to alterations of the material properties of liquid–liquid phase‐separated droplets and affect biochemical enzymatic reactions inside cells.

## Results and Discussion

### Reversible Formation of Coacervates within Lipid Vesicles can be Regulated by Tuning pH

**Figure 1:**
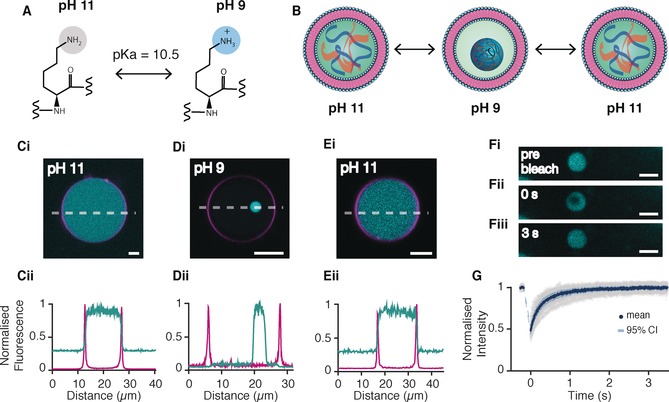
Reversible in situ formation of PLys/ATP coacervates in lipid vesicles by a reduction in pH. A) Polylysine (PLys) switches between a cationic polymer to an uncharged polymer at its p*K*_a_ of pH 10.5. B) Cartoon depicting the pH‐controlled formation of coacervate microdroplets within giant vesicles. C i–E i) Fluorescent confocal images of GUVs made from POPC/Cholesterol containing PLys and ATP at a 4:1 molar ratio. Scale bar=5 μm. C) At pH 11, after washing the outer solution with iso‐osmolar pH 11 buffer solution, D) at pH 9, after the addition of iso‐osmolar pH 7.3 buffer, and E) after returning the pH to pH 11. C ii–E ii) Corresponding intensity profiles (along the white dashed line) of confocal images of DiD fluorescence (magenta) and FITC‐PLys fluorescence (cyan). Fluorescence intensities were normalized by the maximum intensity. F) FRAP of coacervate microdroplets in lipid vesicles. Confocal fluorescence microscopy images of a PLys/ATP coacervate in a GUV before bleaching (i); at bleaching (*t*=0; ii); and after recovery (3 s; iii). Scale bar=5 μm. G) Corresponding FRAP recovery curves for FITC‐PLys. The raw data (shaded gray), mean (dark blue), and 95 % confidence limit (light blue) from 16 experiments are shown. The recovery profile was fit to a double exponential curve to obtain the fast and slow diffusion coefficients: 2.4±1.4 μm^2^ s^−1^ and 0.4±0.17 μm^2^ s^−1^.

To encapsulate coacervate‐forming components, GUVs were formed using the gel‐assisted swelling methodology^19^ in the presence of polylysine (PLys) with either carboxymethyl‐dextran (CM‐dextran) or adenosine triphosphate (ATP, Figure 1 A and Supporting Information, Figure S1). Coacervation was inhibited during vesicle formation by setting the pH above the p*K*_a_ of PLys (pH 10.5, Figure 1 B). At a pH above the p*K*_a_ of PLys, the coacervate components do not interact with one another as the amine groups are deprotonated and are therefore unavailable for phase separation via electrostatic interactions. The GUVs were composed of a phospholipid, POPC, (1‐palmitoyl‐2‐oleoyl‐sn‐glycero‐3‐phosphocholine), cholesterol, and a small fraction of fluorescent lipid dye, DiD (1,1′‐Dioctadecyl‐3,3,3′,3′‐Tetramethylindodicarbocyanine, 4‐Chlorobenzenesulfonate salt), for visualization of the membrane (see the Materials and Methods section of the Supporting Information for details). Vesicles were formed in the presence of a buffer solution containing HEPES (5 mm), sucrose (180–200 mm), PLys (40 mm), and ATP (10 mm), or PLys (40 mm) and CM‐dextran (10 mm) at pH 11, at room temperature in the dark. In order to visualize the coacervate components, the coacervate mixture was doped with 0.25 % (v/v) FITC‐tagged PLys, or FITC‐tagged CM‐dextran. Fluorescent confocal microscopy images showed a population of lipid vesicles by fluorescence of the lipophilic dye, DiD, with FITC fluorescence distributed both inside and outside of the lipid vesicles from FITC‐tagged PLys (Supporting Information, Figure S2). The vesicles were typically between 2–30 μm in diameter, which is expected of GUVs produced by the gel‐assisted swelling method. To ensure that coacervate microdroplets would only form within the vesicles, the GUV dispersions were washed with an iso‐osmolar glucose solution at pH 11 to remove excess coacervate components from the outside of the vesicles (see the Materials and Methods section of the Supporting Information for details). Confocal microscopy images showed that the vesicles had been successfully washed as fluorescence intensity line profiles across the lipid vesicle, normalized to 1, showed a low level of fluorescence intensity on the outside of the lipid vesicles compared to before the wash step (Figure 1 C i,ii and Supporting Information, Figure S2).

Next, we aimed to induce coacervation within the GUVs by lowering the pH below the p*K*_a_ of PLys. The pH of the system was reduced to 9–8.5 by adding an iso‐osmolar glucose buffer at pH 7.3. The final pH was confirmed by undertaking control experiments without GUVs where the same volume of pH 7.3 buffer was exchanged for pH 11 buffer and the pH measured. Confocal fluorescence images following the pH reduction showed initial formation of a number of small droplets inside the lipid vesicles with high FITC fluorescence intensity attributed to the accumulation of FITC‐tagged PLys (Supporting Information, Figure S3). These droplets fused together over time to produce a single highly fluorescent microdroplet within each individual lipid vesicle (Figure 1 D i,ii and Supporting Information, Figure S3). This process of nucleation and growth is characteristic of coacervate formation within lipid vesicles or within w/o emulsions^7^, ^8^ and occurred on the order of tens of minutes (Supporting Information, Figure S3). It is interesting to note that line profiles show no fluorescence intensity from the lipid dye, DiD, within the PLys‐rich droplets and no FITC fluorescence within the lipid membrane (Figure 1 D i,ii). This shows that the coacervate microdroplet is a distinct and separate sub‐compartment within the lipid vesicle as there is no cross‐contamination of the lipid within the coacervates. After formation, the PLys‐rich droplets and lipid vesicles remain stable for at least one day (Supporting Information, Figure S4).

We next aimed to test whether coacervation within GUVs is reversible by increasing the pH to above the p*K*_a_ of pLys. At this pH the amine group is deprotonated and electrostatic interactions between the coacervate components are annulled (Figure 1 A). To do this, we increased the pH by exchanging the outer vesicular solution for an iso‐osmolar glucose buffer at pH 11 and observed that the coacervation process within the GUVs is completely reversible. Confocal fluorescence images show a transition of the FITC fluorescence from a heterogeneous coacervate droplet, to a homogeneous distribution throughout the interior of the GUVs upon increasing the pH of the dispersion to pH 11 as observed prior to the first pH switch (Figure 1 E i,ii and Supporting Information, Figure S5).

To confirm that PLys‐rich droplets, at pH 9, were indeed fluid coacervate microdroplets, fluorescence recovery after photobleaching (FRAP) of FITC‐tagged PLys within the microdroplets in the hybrid protocells was undertaken (Figure 1 F and Supporting Information, Figure S6). FRAP data was normalized for bleaching and the whole coacervate droplet showed a 100 % fluorescence recovery, which is characteristic of the microdroplets and attributed to the liquid‐like and dynamic behavior of coacervates.^20^, ^21^ Furthermore, fitting the recovery profile to a double exponential gave two time constants, *τ*, of 0.19±0.12 s and 3.07±6.6 s and diffusion coefficients of 2.4±1.4 μm s^−1^ and 0.4±0.17 μm s^−1^, respectively (Figure 1 G; see the Materials and Methods section of the Supporting Information for more details). The FRAP recovery and the measured diffusion coefficients are on the same order of magnitude as those obtained from coacervate microdroplets formed in the absence of lipid vesicles. This confirmed that the microdroplets formed within the GUVs triggered by changes in pH are indeed characteristic coacervate droplets.^20^, ^21^, ^22^

To further validate that coacervation was taking place between the PLys and its counter charged electrolyte as described, we undertook the same encapsulation and pH switch methodology but with PLys alone (in the absence of ATP) within the lipid vesicle. At pH 11 the PLys was diffuse and evenly distributed throughout the GUV. Upon acidification to pH 9, there was no change in the distribution of dye (Supporting Information, Figure S7). This data confirmed that the liquid droplets formed via electrostatic interactions and subsequent phase separation between PLys and its counter molecule (ATP).

Taken together, these results demonstrate that our methodology can be used to reversibly tune the formation and dissolution of a single PLys/ATP coacervate microdroplet within a GUV. The fact that these hybrid protocells are stable for at least a day suggests that these platforms can also be used to support enzymatic reactions.

### Dynamic Compartmentalization Facilitates Enzymatic Activity

**Figure 2:**
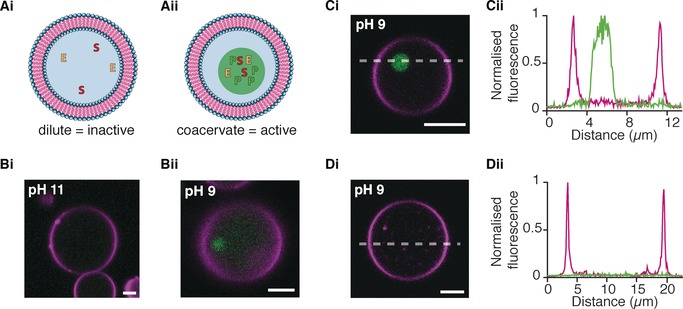
Activation of formate dehydrogenase enzymatic reaction in lipid vesicles through pH‐triggered coacervation. A) Schematics depicting activation of an enzyme by in situ coacervation which leads to an increase in local concentration in the membrane‐free droplet, E: enzyme, S: substrate, and P: Product. A i) A low concentration of enzyme means that the reaction is too dilute and no activity is observed. A ii) In the presence of a coacervate the enzyme and substrates are concentrated into the coacervate and the reaction is initiated. B) Fluorescent confocal microscopy images showing the activity of formate dehydrogenase at 0.1 U mL^−1^ in GUVs upon coacervation at pH 9. B i) No NADH fluorescence is observed within the GUV containing PLys/ATP at pH 11 (magenta). B ii) After switching the pH to 9 and 24 h of incubation at room temperature, fluorescence from NADH (green) was observed within the PLys/ATP coacervate and in the surrounding aqueous solution within the lipid vesicle (magenta). Scale bar=5 μm. C,D) At a low concentration of formate dehydrogenase (0.005 U mL^−1^), sodium formate (5 mm), and *β*‐NAD^+^ (0.45 μm), the enzyme is active only in the presence of a coacervate and subsequent concentration increase. Confocal microscopy images (i) and corresponding line profiles (ii) after 24 hours of incubation at room temperature. C) GUVs (magenta) containing PLys/ ATP and the enzyme show increased NADH fluorescence (green) within the coacervate droplet. D) GUVs without PLys/ ATP coacervates showed no NADH fluorescence at pH 9. Both GUVs were treated with the same pH‐switching methodology as previously described. Scale bar=5 μm. Fluorescence emission of DiD dye within GUV membranes is colored magenta and the autofluorescence of NADH is colored green.

To determine whether in situ dynamic compartmentalization could support and tune enzymatic reactions, we examined the activity of formate dehydrogenase in our system. In this well‐established assay, formate dehydrogenase oxidizes formate to carbon dioxide, with the concatenate reduction of *β*‐NAD^+^ to fluorescent NADH. It has previously been shown that coacervate microdroplets will partition and concentrate a range of substrates and molecules.^23^, ^24^ Therefore, we wondered whether inducing coacervation could alter enzymatic reactions via the concentration of enzyme reactants within the coacervate droplet, as previously observed with aqueous two phase systems^25^ (Figure 2 A).

Firstly, we probed the effect of in situ compartmentalization on the enzyme formate dehydrogenase. FITC‐labelled formate dehydrogenase (0.1 U mL^−1^) was co‐encapsulated within POPC/cholesterol vesicles and the coacervate components at pH 11, as previously described. On reducing the pH to pH 9, we observe a change in the distribution of dye within the GUVs from a homogeneous distribution throughout the interior of the vesicle to a heterogeneous distribution within the GUV by confocal microscopy. There was a region of high fluorescence intensity associated with the coacervate microdroplet, with no observable fluorescence intensity in the aqueous media surrounding the coacervate within the lipid vesicle (Supporting Information, Figure S8). Our results are in agreement with previous studies^8^, ^23^ where enzymes and other molecules partition and concentrate into preformed coacervate droplets. This shows that molecular encapsulation within lipid vesicles, washing, and consequent pH changes does not alter the ability for coacervates to incorporate client molecules, such as enzymes. To ensure that formate dehydrogenase was active after the pH switch, dynamic hybrid protocells were produced as discussed previously but with the addition of the formate dehydrogenase (0.1 U mL^−1^); the substrate, formate (5 mm); and the cofactor, *β*‐NAD^+^ (0.45 mm). Confocal images after 24 h show NADH fluorescence intensity distributed throughout the GUV with an increase of fluorescence intensity within the GUV and the coacervate microdroplet (Figure 2 B i,ii). Our results, along with control experiments (Supporting Information, Figure S9), confirm that formate dehydrogenase is active at pH 9 and after a pH switch.

Building on our results, we wanted to exploit in situ coacervation as a means to activate the formate dehydrogenase reaction via the concentration and change to the local environment of the reactants into the coacervate droplet (Figure 2 A). Control experiments had shown that NADH production within lipid vesicles, in the absence of coacervate‐forming components, was dependent on the concentration of formate dehydrogenase. End‐point measurements showed that at low concentrations of formate dehydrogenase (0.005 U mL^−1^) there was no NADH production, after 24 h, compared to increased concentrations of formate dehydrogenase (0.1, 0.05 U mL^−1^; Supporting Information, Figure S10). We therefore encapsulated formate dehydrogenase (0.005 U mL^−1^) with formate (5 mm) and *β*‐NAD^+^ (0.45 mm) into POPC/cholesterol vesicles with and without PLys and ATP, at pH 11 to determine whether the increase in concentration of formate dehydrogenase into the coacervate droplet would switch on the production of NADH. Following our established methodology, the two populations of vesicles were washed and coacervation was triggered by a reduction in pH to pH 9. After 24 hours, vesicles with and without coacervates were characterized for NADH production. Confocal fluorescence microscopy images showed high fluorescence intensity associated with NADH within the coacervate droplet encapsulated within the vesicle but no NADH fluorescence within the vesicles without coacervate microdroplets (Figure 2 C,D). The data suggests that pH‐triggered coacervation within lipid vesicles can turn on enzymatic reactions by the concentration of materials into the coacervate reaction center, which also leads to changes in the local environment of the enzyme and reactants. Despite this, we wanted to rule out the possibility of enzyme leakage from the interior of the lipid vesicle. If formate dehydrogenase was leaving the coacervate droplet then the production of NADH within the vesicle would be reduced. Control experiments of encapsulated formate dehydrogenase within GUVs showed no decrease in fluorescence from FITC‐tagged formate dehydrogenase within the lipid vesicle (Supporting Information, Figure S11). This supports our observations that NADH was produced by the increased local concentration of the enzyme assay by in situ compartmentalization.

To confirm that coacervate microdroplets are able to switch on reactions by increasing the local concentration and changing the local environment of the enzyme, we compared the production of NADH as a function of time in coacervate dispersions and in buffer at the same molecular concentrations, in the absence of lipid vesicles. To do this, solutions comprised of formate dehydrogenase (0.002 U mL^−1^), formate (25 mm), and *β*‐NAD^+^ (0.6 mm) were prepared with and without PLys and ATP (40 mm and 10 mm, respectively) in HEPES buffer (5 mm) at pH 11. The reaction mixtures were incubated for 30 minutes at pH 11, after which the pH was switched to 9 and NADH production was measured using fluorescence spectroscopy (Supporting Information, Figure S12). Our results show that NADH was produced within the coacervate dispersions but not in the buffer solution. These results confirm that at low concentrations of formate dehydrogenase, coacervate droplets can switch on enzymatic activity by increasing the local concentration. Taken together, our results show that pH‐triggered coacervation within lipid vesicles can activate dormant enzymatic reaction through the concentration and changes to the local environment of molecules in the coacervate reaction center.

### The Formation of Hybrid Protocells is Robust.

**Figure 3:**
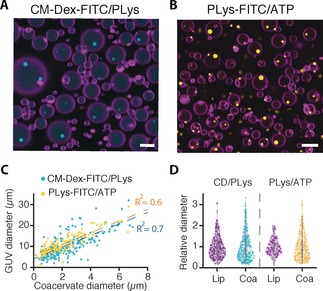
Size characterization of PLys/ATP and CM‐dextran/PLys coacervates formed in GUVs. A) Fluorescence confocal cross‐sections of lipid vesicles containing CM‐dextran/PLys coacervates with FITC‐tagged CM‐dextran (0.5 % (v/v). B) Confocal cross‐sections of GUVs containing PLys/ATP coacervates with FITC‐tagged PLys (0.25 % v/v) at pH 9. Scale bars=10 μm. Fluorescence emission of DiD dye within GUV membranes is colored magenta, FITC‐tagged PLys within CM‐Dex/PLys coacervates is colored cyan and FITC‐tagged PLys in PLys/ATP coacervates is colored yellow. C) Scatter plot of coacervate diameters plotted against vesicle diameters. Data shows a correlation between the size of the vesicle and the internal coacervate. Straight lines fitted to the data and gave R^2^ values of 0.6 (PLys/ATP) and 0.7 (CM‐dextran/PLys). D) Violin plot of the relative diameters of each population show that the relative spread in size variation is similar for both lipid vesicles and coacervates, and between the two populations, when normalized for the mean size.

Having shown that in situ coacervation by pH switching can activate the formate dehydrogenase reaction via a local concentration increase, we assessed the reproducibility of our protocol for the in situ coacervation in lipid vesicles for both PLys/ATP and CM‐dextran/PLys coacervates. The methodology is readily transferrable to other coacervate forming systems, such as CM‐dextran/PLys (Supporting Information, Figure S13) by exploiting the inherent pH responsiveness of PLys. Image analysis of confocal cross‐sections of GUVs encapsulating PLys/ATP or CM‐dextran/PLys droplets was undertaken by using a custom‐written image analysis routine in FIJI. Segmentation of hundreds of lipid vesicles and coacervates (Figure 3 A) that had been generated by a change in pH showed that 17 and 40 % of the GUVs were occupied by coacervates in the case of CM‐dextran coacervates (obtained by image analysis from two repeat experiments; Figure 3 A and Supporting Information, Figure S15) and 24 % in the case of ATP‐based coacervates (Figure 3 B).

Determination of the vesicle and droplet diameters was carried out using FIJI image analysis, only for the GUVs that contained coacervate droplets. The diameters of lipid vesicles varied from 2–30 μm with the diameters of the coacervates ranging from 0.2–8 μm. For the CM‐dextran‐based system *d*_GUV_=10.4±5.4 μm, *d*_CM‐Dextran/PLys_=2.3±1.5, *n*>300 (Supporting Information, Figure S14 A) and for the ATP‐based coacervates the mean diameter and standard deviation was *d*_GUV_=11.2±4.2 μm, *d*_PLys/ATP_=2.1±1.3 μm, *n*>120 (Supporting Information, Figure S13 B). The relative standard deviations (RSD) were comparable between the two systems where the CM‐dextran system varied by 65 % and 52 % for the vesicles and the coacervates, respectively, and for the ATP‐based system the vesicles and coacervates had a coefficient of variation of 62 % and 38 %, respectively.

For those GUVs that contained coacervates, the R^2^ value from a linear fit of the vesicle diameter vs. the coacervate diameter were 0.8 and 0.7 for the CM‐dextran/PLys (obtained from image analysis from two repeat experiments) and 0.6 for the PLys/ATP systems (Figure 3 C and Supporting Information, Figure S15). These results show that for those lipid vesicles that do encapsulate the polymers there is a correlation between the size of the coacervate droplets and the size of the GUVs. This is most likely attributed to the vesicle volume, which reflects the amount of material encapsulated and therefore the size of the coacervate. Furthermore, the variance in the populations of the vesicles and coacervates were compared by normalizing the diameters to the mean diameter of the population. The violin plots show that the spread of data between lipid vesicles sizes and their coacervates was very similar for both the PLys/ATP and CM‐dextran/PLys system (Figure 3 D).

The apparent low encapsulation of less than 50 % of pH‐triggered coacervates within lipid vesicles could be attributed to out‐of‐plane coacervates that were not captured in confocal cross‐sections. Therefore, additional experiments were undertaken to capture the full *z*‐volume of the lipid vesicles using a spinning disk confocal microscope to generate maximum projections of lipid vesicles with PLys/ATP coacervates (see the Materials and Methods and Figure S16 in the Supporting Information). The results showed the presence of coacervates in 100 % of GUVs. Furthermore, the size analysis of maximum projections of lipid vesicles and coacervates gave the same diameters, within error, compared to analysis obtained from confocal cross‐sections (Supporting Information, Figure S16).

Taken together, our analysis shows our methodology is reproducible and applicable to different coacervate systems provided one of the two components has a pH‐dependent moiety. Furthermore, the size of the coacervate droplet is directly influenced by the size of the encapsulating vesicle. Despite the clear reproducibility of the methodology, we sought to improve upon the size distribution of the vesicle and corresponding coacervate droplets by using microfluidic techniques.

### Microfluidic Production of Hybrid Protocells

**Figure 4:**
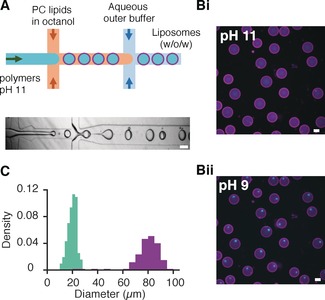
Formation of pH‐triggered coacervation in GUVs formed using microfluidics. A) A double cross‐junction device was used to produce egg PC lipid vesicles containing diffuse PLys and ATP (4:1 molar ratio) at pH 11. The corresponding brightfield image of lipid vesicle production at the two junctions is also shown. Scale bar=100 μm. B) Fluorescence widefield microscopy images showing i) GUVs containing PLys and ATP at pH 11. Cyan fluorescence from FITC‐tagged PLys (0.25 %) and magenta from Texas Red DHPE membrane dye (0.1 %). ii) GUVs containing coacervate microdroplets after a reduction in pH to pH 9, after 15 h. The concentrated FITC‐PLys fluorescence is indicative of the formation of coacervate droplets. Scale bar=50 μm. C) Size quantification of the lipid vesicles (mean diameter=80 μm±12 μm, *n*>220) and their encapsulated coacervates (mean diameter=20 μm±12 μm, *n*>220). 100 % encapsulation efficiency was achieved.

Microfluidic methodologies would enable us to produce monodisperse vesicles encapsulating coacervates with higher reproducibility and lower variance in size in a high throughput manner. Furthermore, a microfluidic approach uses distinct solutions inside and outside the GUVs during formation, which then removes subsequent wash steps required in our bulk methodology. We used double‐emulsion‐based microfluidics to generate lipid vesicles that contain dissolved coacervate components at pH 11. The device was made from PDMS using standard soft lithography methods and had a double cross‐junction geometry^26^ (see the Materials and Methods section in the Supporting Information for details; Figure 4 A i,ii) with channel heights of 50 μm.

Sucrose/HEPES buffer containing diffuse PLys and ATP at pH 11 were flown through the inner aqueous channel to generate w/o emulsions with egg PC lipids dissolved in 1‐octanol.

The first junction (width 50 μm) with a constricted opening facilitates a seamless pinching‐off process to generate w/o droplets stabilized by egg PC and pluronic acid.

The w/o droplets containing non‐coacervated PLys and ATP were converted to a water‐in‐oil‐in water (w/o/w) double‐emulsion droplets at the second junction, which is surface modified and has an aqueous fluid channel of 150 μm in width (see the Materials and Methods section in the Supporting Information). This assists in efficient wetting of the second junction by co‐flowing with the outer aqueous solution of the glucose/HEPES buffer at pH 11 (Figure 4 B i). Coacervation was triggered with the addition of iso‐osmotic pH 7.3 glucose buffer (Figure 4 B ii). Coacervates formed over a 15 h period (Supporting Information, Figure S17). Using this device, we achieved a 100 % encapsulation efficiency (Supporting Information, Figure S18), which has not been possible using other microfluidic approaches.^7^ The lipid vesicles produced were larger and more homogeneous in size compared to the swelling methodology (*d*_GUV_=79.1±11.9 μm) with a RSD of 15 %. The size variation of the coacervate droplets was also reduced compared to bulk methodologies (RSD>50 %), with the *d_coac_*=20.0±3.4 μm and a RSD of 17 % (Figure 4 C) as expected by microfluidic techniques. We have therefore shown that in situ coacervation can be triggered in GUVs prepared by droplet‐based microfluidics by an external change in pH. The applicability of the pH switch in droplet‐based microfluidics demonstrates the use of the pH trigger across multiple experimental set ups.

## Conclusion

In conclusion, we have presented a robust and reproducible methodology for the tunable formation of liquid–liquid phase‐separated droplets within giant unilamellar vesicles. By exploiting the intrinsic p*K*_a_ of cationic PLys we have generated a responsive system where phase separation is triggered though an external reduction in pH. We have further shown that we can utilize the sequestration properties of coacervate droplets for the dynamic in situ activation of enzymatic reactions. This represents a synthetic model for understanding the role of dynamic membrane‐free sub‐compartmentalization in biological systems. Furthermore, our methodology is reproducible using bulk and microfluidic methodologies. Overall our system demonstrates a step forward in the design of multi‐compartment synthetic cells that are dynamically responsive to external stimuli. Such a dynamic system will be of interest in the development of more complex synthetic cells and as minimal models for liquid–liquid phase separation in biological systems.

## Conflict of interest

The authors declare no conflict of interest.

## Supporting information

As a service to our authors and readers, this journal provides supporting information supplied by the authors. Such materials are peer reviewed and may be re‐organized for online delivery, but are not copy‐edited or typeset. Technical support issues arising from supporting information (other than missing files) should be addressed to the authors.

Supplementary
