# Supplementary material for: Reversible pH‐Responsive Coacervate Formation in Lipid Vesicles Activates Dormant Enzymatic Reactions
Source: Angew Chem Int Ed Engl. 2020 Feb 26;59(15):5950–7. doi: 10.1002/anie.201914893 (PMC7187140; doi:10.1002/anie.201914893)
Supplement: Supplementary file 1 — Supplementary [file ANIE-59-5950-s001.pdf]

Angewandte Chemie
Eine Zeitschrift der Gesellschaft Deutscher Chemiker

Supporting Information

# Reversible pH-Responsive Coacervate Formation in Lipid Vesicles Activates Dormant Enzymatic Reactions

*Celina Love, Jan Steinkühler, David T. Gonzales, Naresh Yandrapalli, Tom Robinson, Rumiana Dimova, and T.-Y. Dora Tang**

anie_201914893_sm_miscellaneous_information.pdf

## Author Contributions

C.L. Data curation: Lead; Formal analysis: Lead; Investigation: Lead; Methodology: Lead; Validation: Lead; Visualization: Lead; Writing - Original Draft: Equal; Writing - Review & Editing: Equal
J.S. Investigation: Supporting; Methodology: Equal; Writing - Review & Editing: Equal
D.G. Investigation: Supporting; Methodology: Supporting; Resources: Supporting; Writing - Review & Editing: Equal
N.Y. Methodology: Supporting; Resources: Supporting; Writing - Review & Editing: Supporting
T.R. Funding acquisition: Supporting; Methodology: Supporting; Resources: Supporting; Supervision: Supporting; Writing - Review & Editing: Equal
R.D. Conceptualization: Supporting; Funding acquisition: Supporting; Resources: Supporting; Supervision: Supporting; Writing - Review & Editing: Equal
T.T. Conceptualization: Lead; Data curation: Equal; Funding acquisition: Lead; Investigation: Supporting; Project administration: Lead; Resources: Lead; Supervision: Lead; Writing - Original Draft: Lead; Writing - Review & Editing: Lead.

## Table of Contents

1. Experimental procedures 2

1.1. General reagents 2
1.2. Inner and outer solutions for swelling of giant unilamellar vesicles
1.3. Preparation of lipid vesicle with encapsulated coacervate polymers 2
1.4. Reversible *in situ* formation of coacervates within lipid vesicles triggered by pH 3
1.5. Preparation of FITC tagged enzymes 3
1.6. Activity of formate dehydrogenase in giant unilamellar vesicles 3
1.7. Activity of formate dehydrogase in buffer at different pH 3
1.8. Activity of formate dehydrogenase in bulk coacervate dispersions 3
1.9. Optical imaging of lipid vesicles 3
1.10. Fluorescence recovery after photo bleaching 4
1.11. Microfluidics 4

**2.** Supplementary figures

## 1. Experimental procedures

### 1.1. General reagents

Poly-L-lysine hydrobromide (PLys, $(C_6H_{12}N_2O)_n$, 4 – 15 kDa, monomer MW = 208.1 g mol$^{-1}$), carboxymethyl-dextran sodium salt (CM-Dex, $(C_6H_{10}O_5)_n$.(COOH), 10-20 kDa, monomer MW = 191.3 g mol$^{-1}$), FITC-CM Dextran ($(C_6H_{10}O_5)_n$.(COOH).($C_{21}H_{11}NO_5S$), 4,000 g mol$^{-1}$), adenosine 5-triphosphate disodium salt hydrate (ATP, $C_{10}H_{14}N_5Na_2O_{13}P_3$, 551.1 g mol$^{-1}$), ß-nicotinamide adenine dinucleotide hydrate ($\beta$-NAD$^+$, $C_{21}H_{27}N_7O_{14}P_2$, 663.4 g mol$^{-1}$), sodium formate (HCOONa, 68.0 g mol$^{-1}$), formate dehydrogenase from *Candida boidinii* (FDH, 74 kDa), polyvinyl alcohol (PVA, 89,000 - 98,000 g mol$^{-1}$, 99% hydrolyzed), chloroform ($CHCl_3$, 119.38 g mol$^{-1}$), toluene ($C_6H_5CH_3$, 92.14 g mol$^{-1}$), hydrogen peroxide ($H_2O_2$, 30 wt.%, 34.01 g mol$^{-1}$), hydrochloric acid (37 wt.%, HCl, 36.46 g mol$^{-1}$), poly(sodium 4-styrenesulfonate ($C_9H_7$ $NaO_3S)_n$, 70,000 g mol$^{-1}$), Egg PC (L-α-phosphatidylcholine, 99% TLC, in chloroform, 25 mg mL$^{-1}$, ~768 g mol$^{-1}$), 1-Octanol ($CH_3(CH_2)_7OH$), 130.23 g mol$^{-1}$), Polydiallyldimethylammonium chloride (PDADMAC, $(C_8H_{16}ClN)_n$ 20% wt. in $H_2O$, monomer MW = 161 g mol$^{-1}$), Poly (4-styrenesulfonic acid) (PSS, 18% wt. in $H_2O$, 75000 g mol$^{-1}$) and cholesterol ($C_{27}H_{46}O$, 386.65 g mol$^{-1}$) were all purchased from Sigma Aldrich, Missouri, USA. Fluorescein isothiocyanate isomer I (FITC, $C_{21}H_{11}NO_5S$, 389.38 g mol$^{-1}$), 1,2-Dihexadecanoyl-sn-Glycero-3-Phosphoethanolamine Triethylammonium Salt (Texas Red™ DHPE, $C_{74}H_{117}N_4O_{14}PS_2$, 1 mM, 1381.8 g mol$^{-1}$), DiIC$_{18}$(5) solid(1,1'-Dioctadecyl-3,3,3',3'-Tetramethylindodicarbocyanine, 4 Chlorobenzene-sulfonate Salt (DiD, $C_{67}H_{103}ClN_2O_3S$, 1052.1 g mol$^{-1}$), Pluronic F-63 (Non-ionic surfactant x100, ($C_3H_6O$. $C_2H_4O$)X, ~ 8400 g mol$^{-1}$) and sucrose ($C_{12}H_{22}O_{11}$, 342.3 g mol$^{-1}$) were purchased from ThermoFisher Scientific, Massachusetts, USA. Glucose ($C_6H_{12}O_6$, 180.2 g mol$^{-1}$) and sodium hydroxide (NaOH, 39.997 g mol$^{-1}$) were purchased from Merck, Kenilworth, USA. FITC-PLys ($(C_6H_{12}N_2O)_n$.($C_{21}H_{11}NO_5S$), 25,000 g mol$^{-1}$) was purchased from Nanocs, NewYork, USA. 1-palmitoyl-2-oleoyl-sn-glycero-3-phosphocholine (POPC, $C_{42}H_{82}NO_8P$, 760.1 g mol$^{-1}$) was purchased from Avanti Polar Lipids, Alabama, USA. HEPES ($C_8H_{18}N_2O_4S$, 238.3 g mol$^{-1}$) was purchased from Roth, Karlsruhe, Germany. PDMS (SYLGARD®184 silicone elastomer kit) was purchased from Dow Corning, USA. Osmometer calibration samples (100, 300 mOsmol kg$^{-1}$) were purchased from Gonotec, Berlin, Germany. Picodent Twinsil speed was purchased from Picodent, Wipperfurth, Germany. All materials were used without further purification.

Milli-Q water was used to prepare aqueous stock solutions of PLys (200 mM, pH 8), ATP (100 mM, pH 8), CM-Dextran (1 M, pH 8), FDH (10 U mL$^{-1}$), $\beta$-NAD$^+$ (45 mM), sodium formate (0.5 or 1 M) and HEPES (5 mM, pH 7.3). The pH of all stocks was adjusted using a 10 M NaOH solution. All aqueous stock solutions including buffer were stored at -20 ºC until use. Stock solutions of lipids POPC, Cholesterol and the dye DiD were prepared in chloroform to a concentration of 4 mM and stored under argon at -20 ºC until use. Glass slides were pegylated by leaving the slides in a stirred bath of toluene with PEGsilane (0.2 %), activated with concentrated HCL (0.08 %) overnight at room temperature. Slides were then washed with toluene, ethanol and then water and dried with compressed air and stored in a sealed container.

### 1.2. Inner and outer solutions for swelling of giant unilamellar vesicles (GUVs)

Typical inner liposome solutions consisted of 3.5 - 5 mM HEPES, 180 – 200 mM sucrose and either PLys (40 mM) and ATP (10 mM) or CM-Dextran (40 mM) and PLys (10 mM) with FITC tagged PLys (0.25% v/v). For experiments with the enzyme formate dehydrogenase (Fig. 5), reactants were added to the inner solution to final concentrations of either 0.1 or 0.005 U mL$^{-1}$ (FDH), 4 mM (sodium formate) and 0.45 mM ($\beta$-NAD$^+$). All inner solutions were prepared to pH 11 by addition of 10 mM NaOH. Final osmolarities

ranged between 200 to 240 mOsmol $kg^{-1}$. The osmolarity of the solution was measured with a cryoscopic osmometer, Osmomat 030 (Gonotec, Berlin, Germany) by freezing point depression.

Typical outer solutions consisted of HEPES (2-5 mM) and glucose (200–240 mM) adjusted to either pH 11 (wash solution) or pH 7.3 (trigger solution) with 10 M NaOH solution. The osmolarity was adjusted using either a 2.5 M glucose solution or water, to be within + 5 mOsmol $kg^{-1}$ of the inner solution.

### 1.3. Preparation of GUVs with encapsulated coacervate polymers

Giant vesicles were prepared by hydration of a dried lipid on PVA film as described previously with minor modifications[1]. Glass microscope slides were cleaned with ethanol and water and then dried thoroughly with air. 50 $\mu$L of a 4% (w/v) PVA solution in Mili-Q water was then spread onto three-quarters of a glass microscope slide using a pipette tip and then incubated on a hot plate at 40 $^{\circ}$C for 20 mins to dry the PVA. The temperature of the hot plate was then increased to 55 $^{\circ}$C for another 10 minutes to ensure that the PVA film was completely dry. 2.5 $\mu$L of a 4 mM lipid/chloroform solution containing POPC:Cholesterol:DiD at a molar ratio of 90: 9.7: 0.3 respectively, was then distributed onto the PVA film using a glass Hamiltonian syringe by gently running it over the surface of the PVA. The slide was then left under vacuum for at least 1 hour to remove all of the chloroform.

A chamber was assembled around the dried lipid and PVA film by placing a 1 mm thick Teflon spacer, with a 1 mm diameter hole, on top of the PVA. A cleaned glass cover slip was then placed on top of the spacer and secured with bulldog clips. The chamber was filled with approximately 600 µL of the inner sucrose solution with the coacervate polymers and then left in the dark at room temperature for 30 mins, to allow hydration and swelling of the lipid film and the formation of lipid vesicles. GUVs were harvested by gently tapping the bottom of the chamber, then careful pipetting the solution from the chamber into an Eppendorf tube. Typically, samples were used within 24 hours, but were stable at 4 $^{o}$C for up to a month.

### 1.4. Reversible *in situ* formation of coacervates within lipid vesicles triggered by pH

100 $\mu$L of the lipid vesicle solution was loaded into a chamber formed from an 8 well bottomless $\mu$-Slide with a self-adhesive underside (Ibidi GmbH, Germany) attached to a PEGylated 24 x 60 mm glass cover slips. An additional 500 $\mu$L of the wash solution (pH 11) was added to the GUV dispersion and gently pipetted to mix and then left for 15 mins to allow the lipid vesicles to settle to the bottom of the chamber. An additional 300 µL of wash solution was added and left to equilibrate for 5 mins. The vesicles were then washed by exchanging the chamber solution with 200 $\mu$L of the outer wash solution, at least 6 times, to remove excess coacervate polymers and reactants from the outer solution.

To trigger coacervation within the lipid vesicles, the same volume (typically 200 $\mu$L) of wash solution (pH 11) was exchanged for the trigger solution (pH 7.3), exchange of the buffer was repeated at least twice (400 $\mu$L) and up to four times (800 $\mu$L). To initiate coacervate dissolution, the chamber solution was exchanged for the wash solution (pH 11) in the same way as coacervation formation.

### 1.5. Preparation of FITC tagged enzymes

Proteins were labelled with Fluorescein isothiocyanate (FITC) (10 mM in ethanol) using standard protocols. 10 mg of formate dehydrogenase was dissolved in 0.1 M sodium carbonate buffer (pH 9) at a concentration of 2 mg $mL^{-1}$. Whilst stirring the protein solution, FITC was added stepwise in 5 µL aliquots to a final volume of 20 $\mu$L for every 1 mL of protein solution. The solution was dialyzed for 2 hours in 5 mL of Milli-Q water, and then overnight in another 2 mL of Milli-Q to remove any excess FITC. The tagged dye was stored in aqueous solution at -20 $^{o}$C until use.

### 1.6. Activity of formate dehydrogenase in giant unilamellar vesicles

Following the protocol outlined above, the inner lipid vesicles solution was prepared with formate dehydrogenase, sodium formate and $\beta$-$NAD^+$, to final concentrations of 0.005 U $mL^{-1}$, 5 mM and 0.45 mM respectively, with and without coacervates. GUVs then underwent the same pH triggering methodology *via* washing as described above and microscopy images were obtained immediately before pH switching and 24 h later.

### 1.7. Activity of formate dehydrogenase in buffer at different pH

The enzyme reaction was prepared in 20 $\mu$L of HEPES/sucrose (5 mM/ 200 mM) buffer at pH 7.3. Solutions were adjusted to pH 9 or pH 11 with either 0.2 $\mu$L or 0.5 $\mu$L of a 31.25 mM NaOH solution and loaded into a 384 well plate (microplate,693 PS, Small Volume, LoBase, Med. binding, Black, Greiner Bio-one). Final concentrations of reactants were 0.45 mM ($\beta$-$NAD^+$), 5 mM (sodium formate) and 0.1 U $mL^{-1}$ (formate dehydrogenase). The reaction was initiated by the addition of the enzyme and the fluorescence observed using the TECAN Spark 20M well plate reader by measuring the increase in NADH fluorescence over time ($\lambda_{ex}$ = 340 nm and $\lambda_{em}$ = 460 nm, 5 nm bandwidth at 25 $^{\circ}$C).

### 1.8. Activity of formate dehydrogenase in bulk coacervate dispersions

Enzyme activity was confirmed by observing formate dehydrogenase kinetics in dispersions of coacervate microdroplets. The enzyme reaction was prepared in 20 $\mu L$ of HEPES/Sucrose (5 mM/ 200 mM) buffer or in buffer with PLys:ATP coacervates (final coacervate concentration 40:10 mM). Final concentrations of reactants were 0.45 mM ($\beta$-$NAD^+$), 5 mM (sodium formate) and 0.002 U $mL^{-1}$ (formate dehydrogenase). The reaction was initiated by the addition of the enzyme, mixed, and then loaded (20 $\mu$L) into a

384 well plate (microplate,693 PS, Small Volume, LoBase, Med. binding, Black, Greiner Bio-one). The kinetics of the reactions were recorded using a TECAN Spark 20M well plate reader spectrophotometer (Tecan AG, Männedorf, Switzerland) by measuring the increase in NADH fluorescence as described previously.

### 1.9. Optical imaging of GUVs

Fluorescence confocal microscopy of GUVs was undertaken using a Zeiss LSM 880 Airy inverted laser scanning confocal microscope equipped with a Zeiss 40x/1.2 C-Apochromat DIC water immersion objective, a Zeiss 20x/0.8 Plan-Apochromat air objective and a 32 GaAsP PMT channel spectral detector. FITC labelled coacervates were imaged ($\lambda_{FITC}$ = 488 nm) by the 488 nm laser and emission wavelengths detected at $\lambda_{FITC}$ = 495-579 nm. The DiD labelled lipid membranes ($\lambda_{DID}$ = 644 nm) were excited with a 633 nm laser for excitation and emission wavelengths were detected at $\lambda_{DID}$ = 652-695 nm. NADH, the product of the formate dehydrogenase reaction ($\lambda_{NADH}$ = 340 nm), was excited using a 355 nm DPPS UV laser and emission wavelengths detected at $\lambda_{NADH}$ = 420-500 nm. Characterisation of GUVs and coacervate sizes was carried out with images obtained using the 40x lens. Fiji was used to obtain the fluorescence intensity across the GUVs using the line plot function for both the DiD channels and FITC channels. The fluorescence intensity of each of the channels was normalized by its own maximum intensity, or to the maximum intensity of the DiD channel. The images were further processed using a custom-made Fiji (Image J) macro that segmented GUV membranes and the coacervates inside them and measured their diameters. The data was plot using MATLAB (Mathworks) to determine size of the vesicles or the coacervates. The correlated size distributions were fit to a linear regression model to calculate the $R^2$ values using MATLAB. The diameters were normalized by the mean to obtain the relative diameter.

To further assess the encapsulation efficiency, GUVs with coacervates were also imaged with an Olympus IXplore IX83 inverted spinning disk microscope equipped with a Yokogawa CSU W1 confocal scanning unit, an Orca Flash4 V.3 sCMOS camera and controlled by CellSens software. Images were acquired using an Olympus UPLXAP040x2 40x/0.95 NA air objective and an UPLSAPO100XS 100x/1.35 NA silicone oil objective. GUVs and coacervates were imaged with 640 nm and 488 nm laser lines using a Coherent OBIS laser with band pass filters 685 /40 nm and 525/50 nm respectively (Semrock, IDEX health and science, USA). Z-stacks of GUVs were acquired and reconstructed as maximum projections using FIJI. GUVs and coacervates were segmented manually and the diameters obtained by FIJI. The data was then plotted and fit using Matlab.

### 1.10. Fluorescence recovery after photobleaching

Fluorescence recovery after photobleaching (FRAP) experiments were undertaken for PLys/ATP coacervates microdroplets encapsulated in GUVs. All FRAP experiments were carried out using an Andor Eclipse Ti inverted spinning disk confocal microscope, equipped with a FRAPPA module and an Andor iXON 897 Monochrome EMCCD camera and imaged using a Nikon 60x/1.2 DIC Plan Apochromat VC water immersion objective. Bleaching of the droplet was achieved using a 405 nm diode laser at 100% power. Imaging was carried out using a 488 nm DPSS laser for excitation of the FITC dye at $\lambda_{FITC}$ = 488 nm with an emission wavelength of $\lambda_{FITC}$ = 500-590 nm. Due to the fast recovery of the coacervates, a reduced frame size of 4 x 512 dexels was used so that a higher temporal resolution could be achieved. For each experiment, 20 pre-bleach images were acquired before bleaching. The fluorescence recovery was then recorded by imaging for 3 mins every 4 msec in the FITC channel only.

A custom written FIJI script was used to extract raw fluorescence data which was then normalized against a background region, a reference droplet and by the total fluorescence of the whole bleached droplet[2–4]. Using a custom MATLAB script, recovery profiles were fit to a double exponential curve using Eq. 1 to obtain the time constants $\tau_1$ and $\tau_2$.

$$f(t) = \begin{cases} 1 & t < t_0 \\ 1 - A1\exp\left(-\frac{t-t_{\mathrm{bleach}}}{\tau_1}\right) + A2\exp\left(-\frac{t-t_{\mathrm{bleach}}}{\tau_2}\right) + C & t \geq t_0 \end{cases} \quad \text{Eq.1}$$

where $t_0$ is the first time point after bleaching, A1 and A2 are exponential prefactors, $t$ is the time and $t_{bleach}$ is the time of bleaching. The time constants were calculated from at least 16 bleaching events from at least two different samples. Fitted time constants were then converted to diffusion constants using Eq. 2 [5], where r is the radius of the bleach spot.

$$D = \frac{0.88r^2}{4\tau In2} \quad \text{Eq.2}$$

### 1.10. Microfluidics

Giant vesicles were produced using a microfluidic chip design with two consecutive cross-junctions in a flow-focusing configuration that generates w/o/w double emulsion droplets. This design was based on the work published by Petit et al[6] and fabricated in the Robinson Lab using standard lithography methodologies. Briefly, PDMS and curing agent (Sylgard 184, Dow Corning) were mixed in a 9:1 ratio, degassed for 30 min and poured on top of the silicon wafer to a height of 5 mm in a Petri dish. The PDMS was further degassed for 10 min and cured in an oven at 90 ºC for 3 h. After which the PDMS was left to cool to room temperature and

peeled from the wafer. Inlets were generated by punching holes using 1 mm biopsy puncher (Kai Europe GmbH) and then bonded to glass slides which had been cleaned with ethanol and water. For successful bonding, both the glass coverslips and PDMS slices were treated with an air plasma at 0.6 bar for a period of one minute by plasma (PDC-002-CE, Harrick Plasma). Microfluidic chips were kept on a hot plate at 60 °C for 2h to complete the bonding process before further use. All channels were 50 *μ*m in height. The outer solution channels of the microfluidic chip were hydrophilized by flushing the channels with an oxidizing solution, 3:1 mix of 30 wt.% $H_2O_2$ and 37 wt.% HCl for 5 mins and then treating the channels with 5 vol.% PDADMAC and 2 vol.% poly(sodium 4-styrenesulfonate) for 2 mins with water washes in between.

The microfluidic chips were loaded onto a Zeiss Aziovert 200M inverted widefield microscope equipped with a 16 channel CooLED pE-300-W and ANDOR ZYLA fast sCMOS camera and imaged using 10x air objective (10x/0.3 Plan-Neofluar, Air, Ph1, Zeiss). FITC was imaged using a 470 nm broad spectrum LED for excitation through a GFP/Alexa 488/FITC filter set (excitation bandpass 449-489 nm, dichroic longpass 497 nm, emission bandpass 502-549 nm). Texas Red DHPE in the lipid membrane was imaged using a 550 nm broad spectrum LED for excitation through a ROX filter set (excitation bandpass 575 ± 15 nm, beam splitter HC BS 596, emission bandpass 641 ± 75 nm). The three fluid phases were controlled using three Mitos pressure pumps (Dolomite, Royston, UK). Target pressures were 70-80-100 mbar for the inner-middle-outer solutions respectively. The inner solution and the outer solution have been described previously with the addition of 2 % pluronic in the outer solution at pH 11, the middle solution was an oil phase comprised of EggPC lipids at a final concentration of 6.5 mM dissolved in 1-Octanol with 0.008 mM Texas Red DHPE. Lipids were prepared by drying the required volumes of stock solution of EggPC dissolved in chloroform (25 mg $mL^{-1}$) and Texas RED DHPE dissolved in chloroform (1 mM) in a glass test tube under nitrogen for 15 mins, then left under vacuum for 1 hour. Solutions were the dissolved in 1.5 mL of 1-octanol by sonicating at 37°C in a water bath for 1 hour. Solutions were stored at room temperature and used within 24 hours.

The vesicles were collected into micro-centrifuge tubes after formation and then pipetted onto homemade capillary slides formed from a 22 x 22 mm BSA coated cover slips mounted onto a parafilm channel on a 22 x 75 mm microscope slide. 15 µL of the GUV dispersion were loaded into the chamber, imaged using fluorescence widefield microscopy as described previously. Coacervation was initiated by flushing at least 7 µL of pH 7 buffer into the channel as described previously. The capillary channel was sealed completely with Picodent Twinsil speed curing silicone and images were taken every 5 minutes for 15 hours. Image analysis was undertaken using FIJI and custom written code as described previously.

## 2.0 Supplementary figures

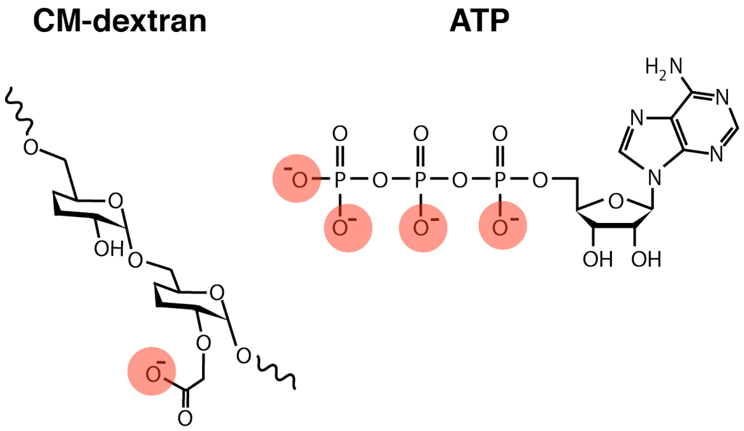


**Figure S1.** Chemical structures of the negatively charged coacervate molecules, carboxymethyl-Dextran (CM-Dextran) and adenosine triphosphate (ATP) that undergo electrostatic attraction and coacervation with positively charged PLys at pH 9.

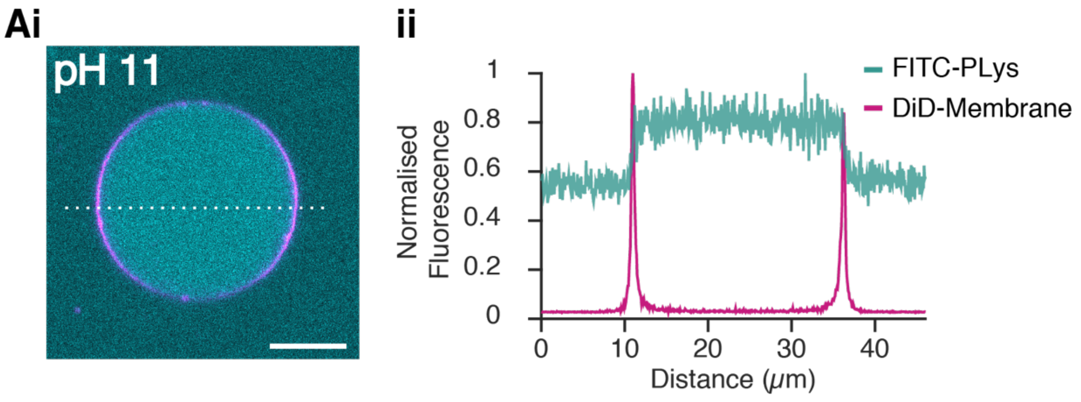


**Figure S2. (Ai)** Confocal microscopy image (cross section) showing POPC/cholesterol lipid vesicles doped with DiD dye (0.3% mol) containing PLys and ATP (40/10mM) doped with 0.25 % FITC-PLys at pH 11 after swelling and harvesting of lipid vesicles before washing. Scale bar = 10 $\mu$m. (ii) Intensity profile of confocal image (dotted white line) showing fluorescence intensity from FITC-PLys both inside and outside of the lipid vesicle.

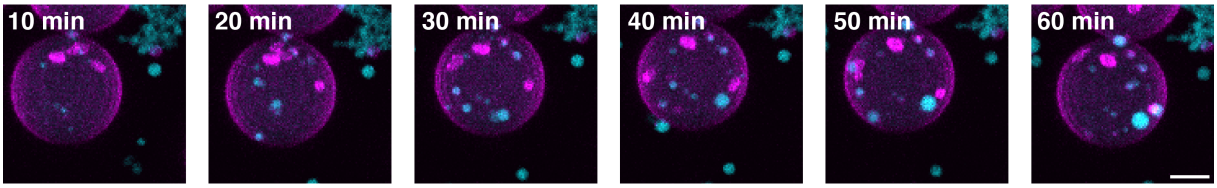


**Figure S3.** Confocal microscopy max projections of the formation of PLys/ATP coacervates (cyan) in washed GUVs (magenta) over 60 mins after changing the pH from pH 11 to pH 9. The images show the same lipid vesicle. Scale bar = 5 $\mu$m.

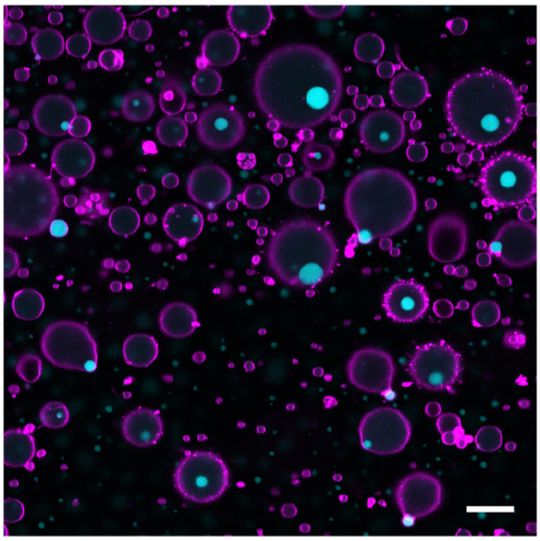

**Figure S4**. GUVs at pH 9 containing CM-Dextran/PLys coacervates 24 hours after undergoing a pH switch from pH 11. Scale bar = 10 $\mu$m.

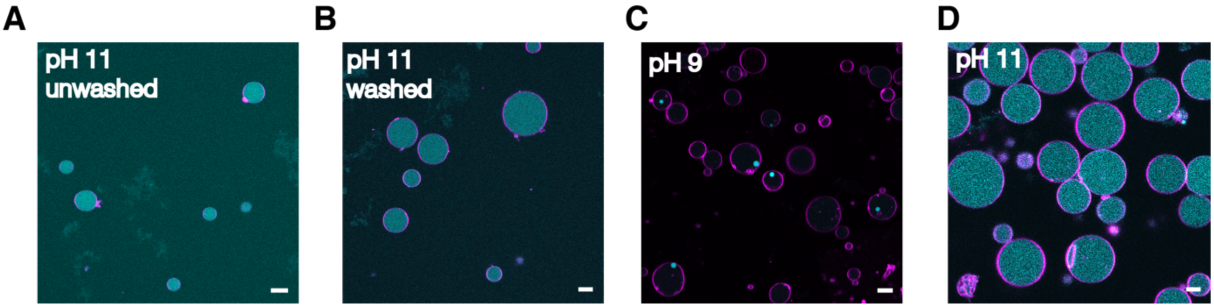


**Figure S5.** Fluorescent confocal microscopy images showing populations of GUVs fluorescently labelled with DiD dye (0.3 mol %) encapsulating PLys/ATP (40 mM/10mM) doped with 0.25% (v/v) FITC-labelled PLys **(A)** at pH 11 after swelling, **(B)** after washing of the lipid vesicles at pH 11, **(C)** after a pH switch to pH 9 and **(D)** after returning the pH to pH 11. Scale bar = 10 $\mu$m.

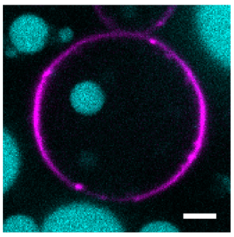

**Figure S6.** Fluorescent confocal microscopy image showing a GUV fluorescently labelled with DiD dye (0.3 mol %) encapsulating PLys/ATP (40 mM/10 mM) as an example for a hybrid cell that underwent the FRAP experiment. Scale bar = 5 $\mu$m.

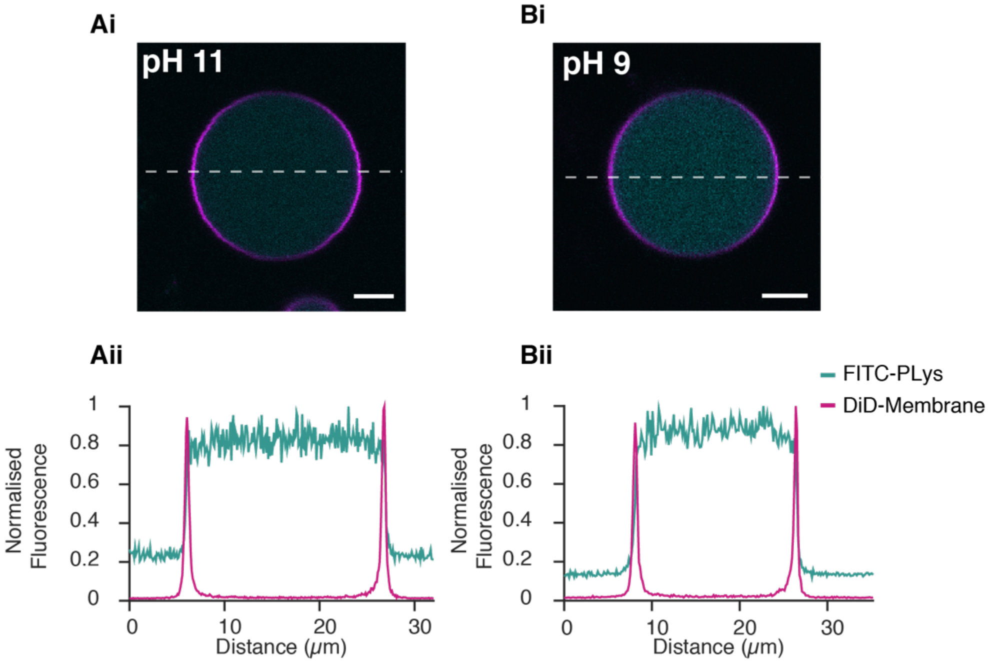


**Figure S7.** Control experiments of GUVs encapsulating PLys doped with 0.25 % FITC labelled PLys, which have been washed at pH 11 and the pH subsequently reduced to pH 9. Confocal optical microscopy images (i) and corresponding intensity profile across the white dotted line (ii) of **(A)** at pH 11 and **(B)** at pH 9. No coacervates form without ATP within the GUVs after acidification. Scale bar = 5 μm.

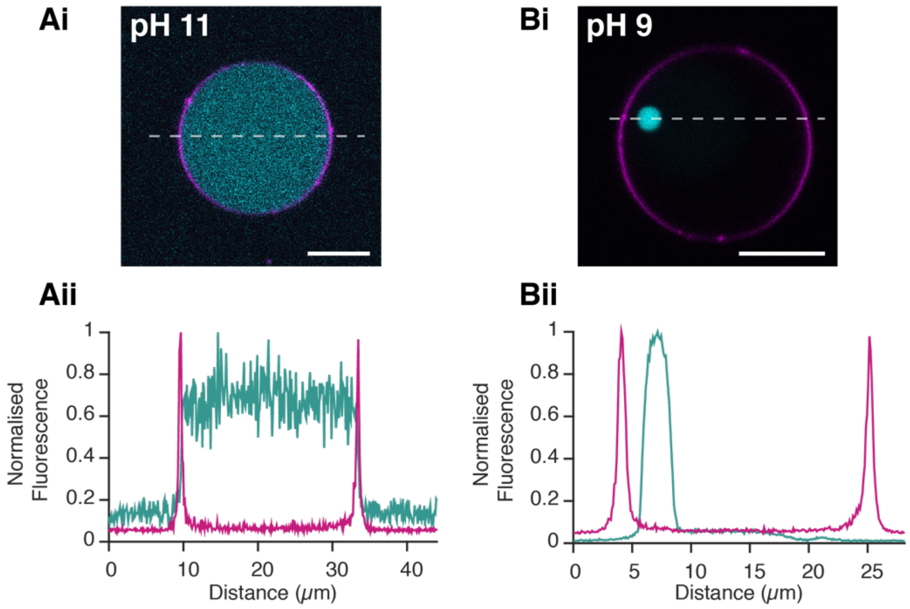


**Figure S8. (A)** Fluorescent confocal microscopy images showing the up-concentration of 0.1 U mL$^{-1}$ of FITC-tagged formate dehydrogenase upon coacervation of CM-Dextran/PLys within a POPC/cholesterol GUV after a pH switch from pH 11 to pH 9 and the corresponding intensity profile **(Aii). (Bi)** Up-concentration of formate dehydrogenase into the coacervate droplets upon coacervation at pH 9. **(Bii)** Corresponding line profile. Scale bar = 5 μm.

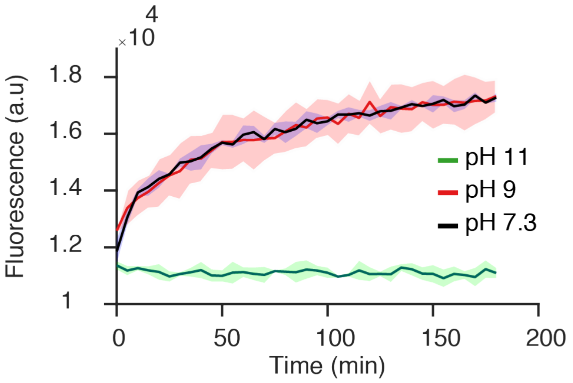


**Figure S9**. Activity of 0.1 U mL$^{-1}$ of formate dehydrogenase with 0.45 mM of *β*-NAD$^+$ and 5 mM of sodium formate in HEPES/sucrose (5 mM/ 200 mM) buffer at pH 7.3, 9 and 11. Activity was obtained by measuring NADH fluorescence. The results show that the enzyme is active at pH 9 and 7.3 and inactive at pH 11. Errors are obtained from three repeat experiments.

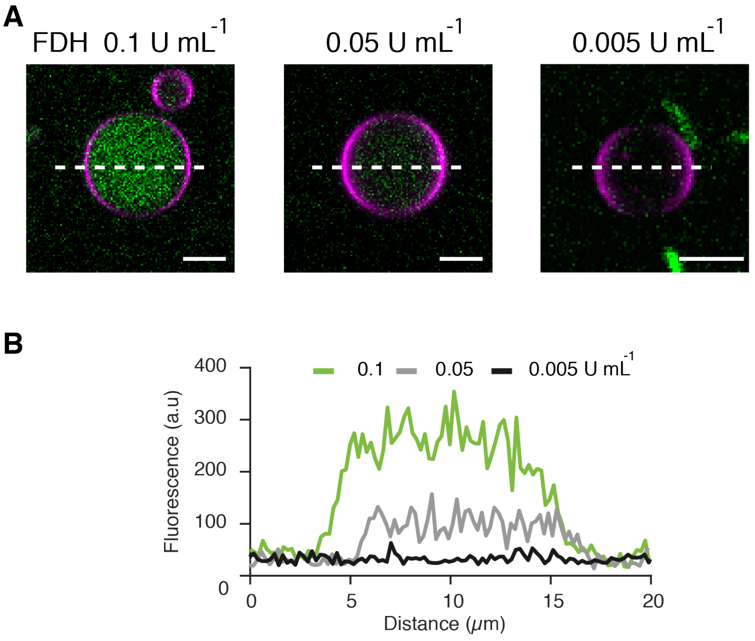


**Figure S10**. NADH production by formate dehydrogenase within POPC/cholesterol GUVs at pH 9. **(A).** Fluorescent confocal microscopy images of NADH fluorescence in GUVs encapsulating *β*-NAD$^+$ (0.45 mM) and formate (5 mM) with decreasing formate dehydrogenase concentration (0.1, 0.05, 0.005 U mL$^{-1}$) after 24 hours of incubation at room temperature. Scale bar = 5 $\mu$m. **(B)** Intensity plots of NADH fluorescence from GUVs containing 0.1 U mL$^{-1}$ (green line), 0.05 U mL$^{-1}$ (grey line), 0.005 U mL$^{-1}$ (black line). NADH was produced at 0.1 U mL$^{-1}$ and 0.05 U mL$^{-1}$ but no appreciable NADH fluorescence at 0.005 U mL$^{-1}$. All confocal images were obtained on the same laser intensity and detector gain settings.

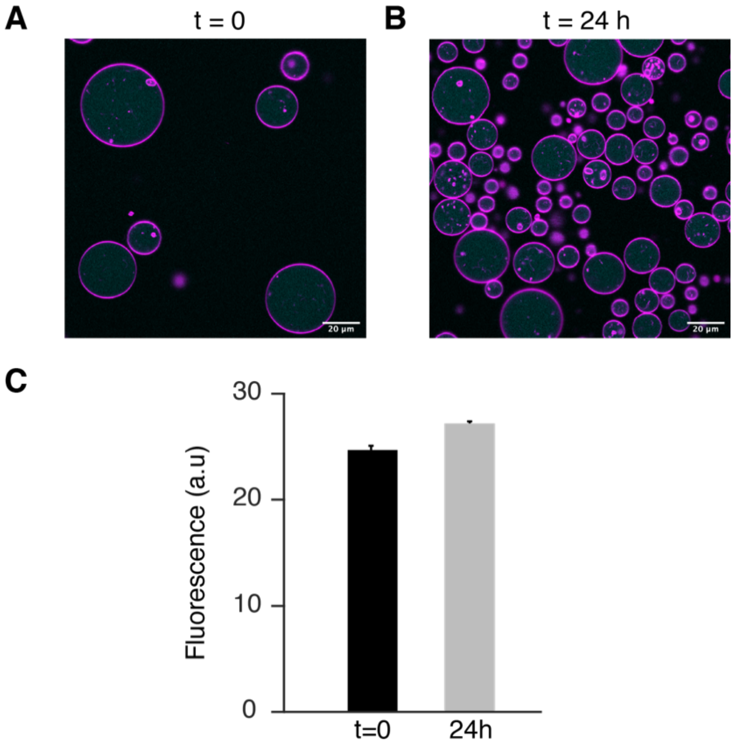


**Figure S11.** Control experiment showing that FITC tagged formate dehydrogenase remains encapsulated within the vesicles after 24 hrs. **(A)** Confocal microscopy images of FITC tagged formate dehydrogenase (0.1 U mL$^{-1}$) encapsulated within in POPC/cholesterol GUVs at pH 9 at t=0 h **(B)** Images of the same vesicle population after 24 hours. **(C)** Mean fluorescence intensity of the FITC tagged formate dehydrogenase within the GUVs at t= 0 and t = 24 h. The data shows no decrease in fluorescence intensity over 24 h. Error bars indicate the standard deviation from at least 20 vesicles.

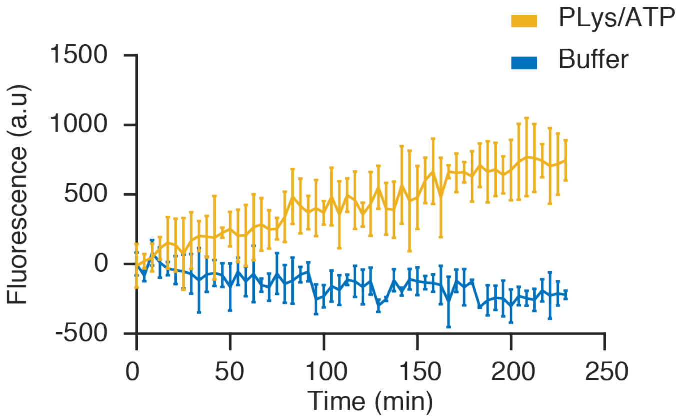


**Figure S12.** Activity of 0.002 U mL$^{-1}$ formate dehydrogenase in PLys/ATP (40 mM/ 10 mM) coacervate dispersions in the absence of vesicles obtained using a TECAN Spark 20 M spectrometer. The coacervate and enzyme dispersions were held at pH 11 for 30 mins before reducing to the pH to pH 9. No activity was observed in the buffer. However, formate dehydrogenase was active within the coacervate dispersion and NADH production increased linearly with time.

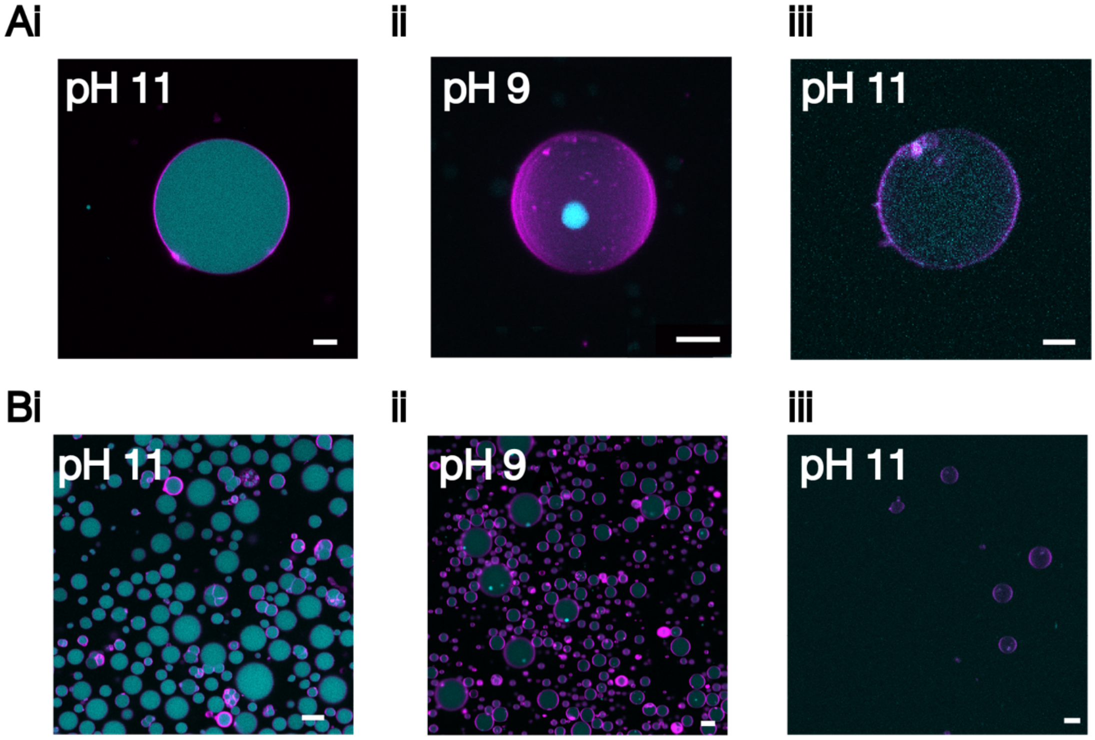


**Figure S13.** Confocal microscopy images of **(A)** single POPC/cholesterol GUVs and **(B)** populations of vesicles containing CM-Dextran/PLys (40 mM/10 mM) **(i)** at pH 11 where the polymers are diffuse, (ii) at pH 9 where coacervates have formed within the vesicles after a pH switch (note that not all GUVs show the presence of coacervate droplets despite 100 % encapsulation at pH 11, Aii shows a maximum projection whilst images in B are confocal cross sections), (iii) after a return to pH 11, the coacervate droplets dissolve. **(A)** Scale bar = 5 $\mu$m **(B)** Scale bar = 10 $\mu$m.

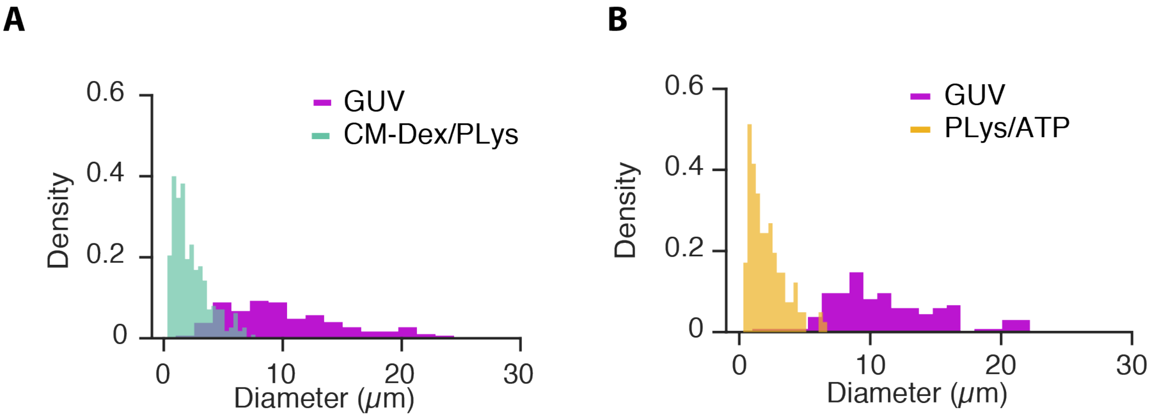


**Figure S14** **(A)** Histogram showing the diameters of the CM-Dextran/PLys coacervates and the corresponding GUVs, n = 128. **(B)** Histogram plotting the diameters of GUVs and PLys/ATP coacervates, n=304.

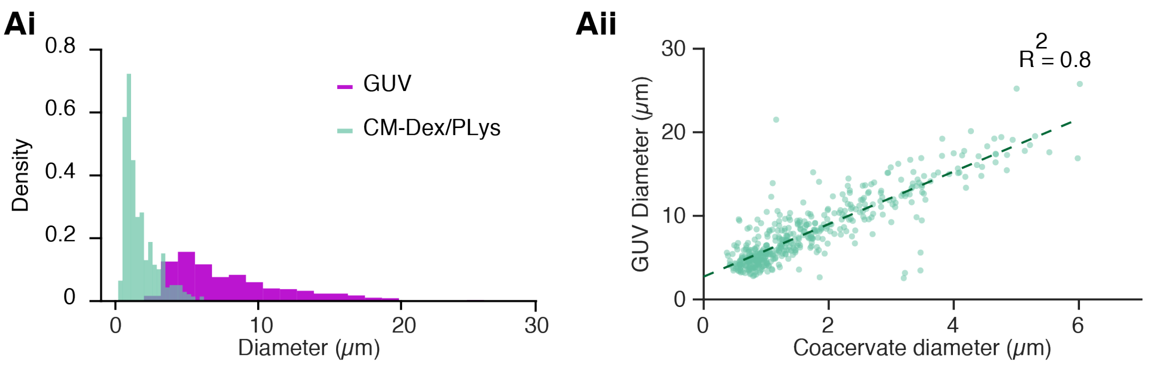


**Figure S15.** Size characterization of CM-Dextran/PLys coacervates formed in GUVs from a repeat experiment. **(Ai).** Histogram showing the quantified diameters of the CM-Dex/PLys coacervates and their encapsulating GUVs. Mean coacervate diameter 1.70 ± 1.14 $\mu$m, mean vesicle diameter 8.06 ± 4.12 $\mu$m, n > 450. **(Aii)** Scatter plot of coacervate diameters plotted against vesicle diameters. Data shows a correlation between the size of the GUV that of the encapsulated coacervate. Straight lines fitted to the data gave similar $R^2$ values of 0.8 compared to other repeat experiments.

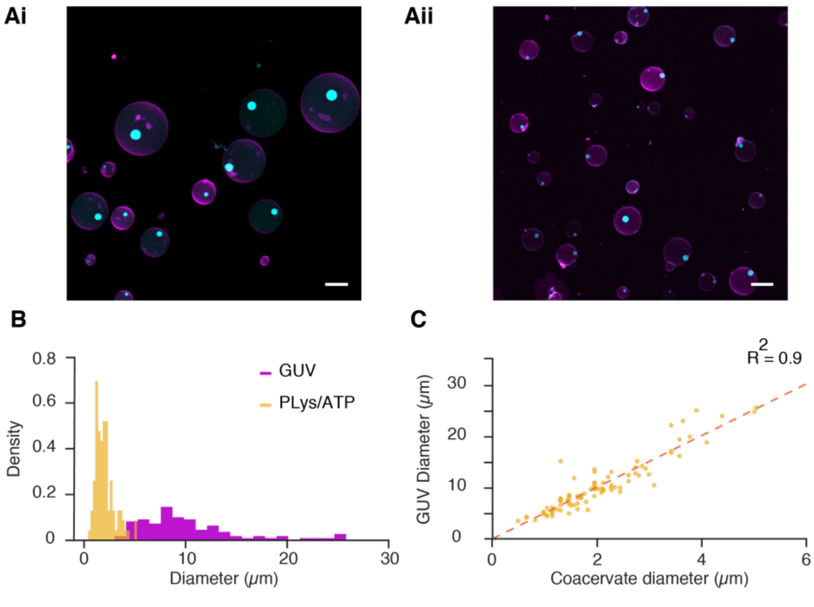


**Figure S16:** Characterization of PLys/ATP coacervates formed in GUVs using a spinning disk microscope to obtain maximum projections from a repeat experiment **(A)** Max projections of GUVs containing PLys/ATP coacervate droplets taken using **(i)** 100 x and **(ii)** 40 x objective show 100 % of the GUVs contained coacervate droplets compared to 17 and 42% from analysis of confocal cross sections. The difference in apparent encapsulation is due to the analysis of confocal cross sections vs maximum projection images which only represent a single plane of view. Scale bar = 10 $\mu$m **B)** Histogram showing the quantified diameters of the coacervates and their encapsulating GUVs. Mean coacervate diameter 2.02 ± 0.96 $\mu$m (RSD = 48 %), mean vesicle diameter 10.25 ± 4.90 $\mu$m (RSD = 48 %), n = 96. **(C)** Scatter plot of coacervate diameters plotted against vesicle diameters. Data shows a correlation between the size of the GUV that of the encapsulated coacervate with an $R^2$ value of 0.9.

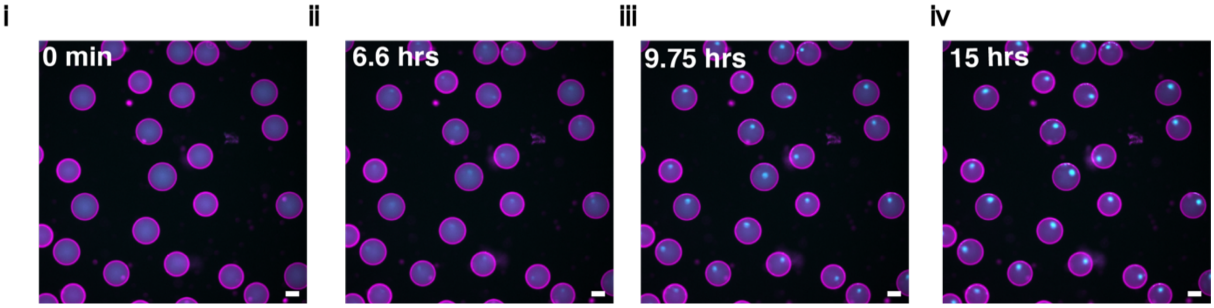


**Figure S17.** Fluorescent confocal microscopy images of Egg PC lipid vesicles doped with Texas Red DHPE (0.1 mol%) and PLys/ ATP coacervates doped with 0.25 % v/v FITC tagged PLys prepared by microfluidic methodologies. The lipid vesicles were prepared at pH 11 and the pH was switched to pH 9. Fluorescence widefield images were obtained at **(i)** 0 mins, **(ii)** 6.6 h, **(iii)** 9.75 h and **(iv)** 15 h. Scale bar = 50 $\mu$m.

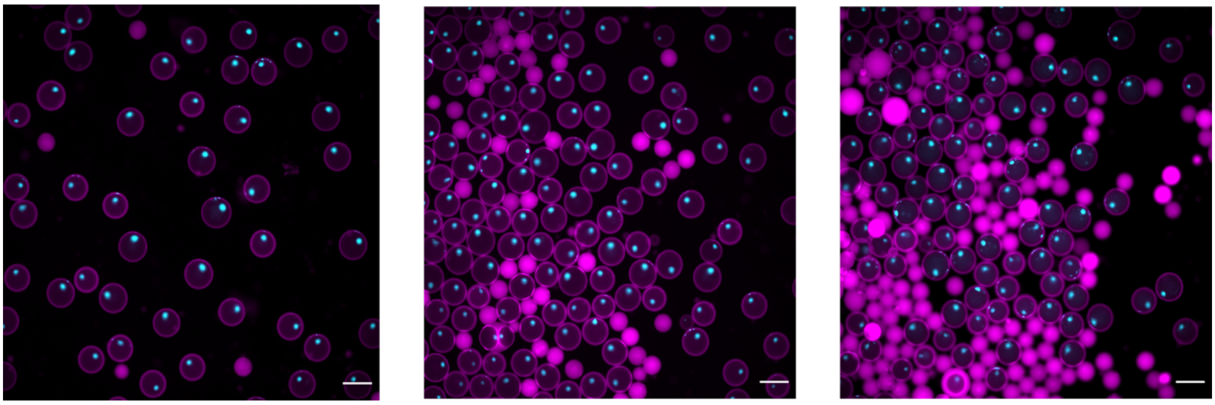

**Figure S18.** Wide field microscopy images showing three regions of interest (from the same experiment) of a population of GUVs prepared using microfluidics, 15 hours after a pH switch from pH 11 to 9. Scale bar = 100 $\mu$m.

## References:

[1] A. Weinberger, F.-C. Tsai, G. H. Koenderink, T. F. Schmidt, R. Itri, W. Meier, T. Schmatko, A. Schröder, C. Marques, *Biophys. J.* **2013**, *105*, 154–64.

[2] R. Eils, C. Kappel, *Confocal Appl. Lett.* **2004**, *18*.

[3] R. D. Phair, S. A. Gorski, T. Misteli, **2003**, pp. 393–414.

[4] B. Drobot, J. M. Iglesias-Artola, K. Le Vay, V. Mayr, M. Kar, M. Kreysing, H. Mutschler, T.-Y. D. Tang, *Nat. Commun.* **2018**, *9*, 3643.

[5] D. Axelrod, D. E. Koppel, J. Schlessinger, E. Elson, W. W. Webb,*Biophys. J.* **1976**, *16*, 1055–1069.

[6] J. Petit, I. Polenz, J.-C. Baret, S. Herminghaus, O. Bäumchen, *Eur. Phys. J. E* **2016**, *39*, 59.

## Author Contributions:

T-Y-D.T. conceived the research and acquired funds. RD and TR contributed funds to the project. CL, JS, DG, RD contributed to the design and the undertaking of the experiments. NY and TR contributed the microfluidic devices. CL analysed the data. CL, JS, T-Y DT contributed to the interpretation of data. CL, T-YDT wrote the original draft of the manuscript and all authors contributed to the final manuscript.
